# Synthesis of PVA/CeO_2_ Based Nanocomposites with Tuned Refractive Index and Reduced Absorption Edge: Structural and Optical Studies

**DOI:** 10.3390/ma14061570

**Published:** 2021-03-23

**Authors:** Shujahadeen B. Aziz, Elham M. A. Dannoun, Dana A. Tahir, Sarkawt A. Hussen, Rebar T. Abdulwahid, Muaffaq M. Nofal, Ranjdar M. Abdullah, Ahang M. Hussein, Iver Brevik

**Affiliations:** 1 Hameed Majid Advanced Polymeric Materials Research Lab., Department of Physics, College of Science, University of Sulaimani, Qlyasan Street, Sulaimani 46001, Kurdistan Regional Government, Iraq; sarkawt.hussen@univsul.edu.iq (S.A.H.); rebar.abdulwahid@univsul.edu.iq (R.T.A.); ranjdar.abdullah@univsul.edu.iq (R.M.A.); ahang.hussein@univsul.edu.iq (A.M.H.); 2 Department of Civil engineering, College of Engineering, Komar University of Science and Technology, Sulaimani 46001, Kurdistan Regional Government, Iraq; 3 Associate Director of General Science Department, Woman Campus, Prince Sultan University, P.O. Box 66833, Riyadh 11586, Saudi Arabia; elhamdannoun1977@gmail.com; 4 Department of Physics, College of Science, University of Halabja, Halabja 46018, Kurdistan Region, Iraq; dana.tahir@uoh.edu.iq; 5 Department of Physics, College of Education, University of Sulaimani, Old Campus, Sulaimani 46001, Iraq; 6 Department of Mathematics and General Sciences, Prince Sultan University, P.O. Box 66833, Riyadh 11586, Saudi Arabia; muaffaqnofal@gmail.com; 7 Department of Energy and Process Engineering, Norwegian University of Science and Technology, N-7491 Trondheim, Norway; iver.h.brevik@ntnu.no

**Keywords:** PVA, CeO_2_ nanoparticle, XRD analysis, UV–Vis investigation, optical properties, bandgap calculation

## Abstract

In the current study, polymer nanocomposites (NCPs) based on poly (vinyl alcohol) (PVA) with altered refractive index and absorption edge were synthesized by means of a solution cast technique. The characterization techniques of UV–Vis spectroscopy and XRD were used to inspect the structural and optical properties of the prepared films. The XRD patterns of the doped samples have shown clear amendments in the structural properties of the PVA host polymer. Various optical parameters were studied to get more insights about the influence of CeO_2_ on optical properties of PVA. On the insertion of CeO_2_ nanoparticles (NPs) into the PVA matrix, the absorption edge was found to move to reduced photon energy sides. It was concluded that the CeO_2_ nanoparticles can be used to tune the refractive index (n) of the host polymer, and it reached up to 1.93 for 7 wt.% of CeO_2_ content. A detailed study of the bandgap (BG) was conducted using two approaches. The outcomes have confirmed the impact of the nanofiller on the BG reduction of the host polymer. The results of the optical BG study highlighted that it is crucial to address the ε” parameter during the BG analysis, and it is considered as a useful tool to specify the type of electronic transitions. Finally, the dispersion region of *n* is conferred in terms of the Wemple–DiDomenico single oscillator model.

## 1. Introduction

Polymer composites are polymers containing modifiers that are utilized as passive and also active layers in optoelectronic devices, such as solar cells, light-emitting diodes (LED), optical waveguides materials, and photochromic materials [1]. In this regard, seeking low-cost photovoltaic materials and minimum energy consumption during the manufacturing process is becoming increasingly demanding [2]. To achieve this level of demand, blending certain fillers with a number of functional polymers (i.e., polar polymers) at the atomic level of interaction has been carried out. For example, using the earlier mentioned methodology, a promising class of inorganic–organic nanostructured (NS) materials, i.e., polymer nanocomposites (NCPs) with excellent mechanical strength can be obtained [3]. Such polymer NCPs with high refractive-index (RI) and optical materials are commonly used in optical waveguides, optical reflectors, glass lenses, and camera lenses. Organic materials have many compensations, such as transparency, light weight, low cost, simple processing, and excellent mechanical properties. These material types, however, still have a typical low refractive index disadvantages [4]. It is known that polymers have dual functions, including reducing and capping agents for nanoparticles (NPs) encompassing, which provides chemical and environmental stability [5,6,7]. It is reported that modifications of the chemical, mechanical, optical, and electrical properties of many polymer matrices can be accomplished by NPs incorporation [8,9,10,11,12,13]. Herein, poly (vinyl alcohol) (PVA) is a water-soluble polymeric material that has interesting optical properties [14]. It exhibits good thermal and chemical stability, relatively high dielectric strength, is hydrophilic and nontoxic, and has sufficient storage capacity and dopants (DPs)-dependent optical and electrical properties [15,16,17,18]. It is of great importance to improve several properties in terms of structure, electrical, and chemical properties. For this purpose, the insertion of compatible DPs materials into the PVA matrix may change the PVA structure that will be suitable for a particular application. It was formerly reported that the alteration in PVA structure with better performance could be completed via the inclusion of proper DPs into polymer chains [18,19,20]. For instance, due to the combination of both polymers and DPs properties, there is an agreement within the scientific community that PVA-based metal oxide NCPs have unique characteristics [19]. The presence of polar groups in hydrophilic polymers makes them firmly coordinated with the fillers’ cation or surface groups, forming homogeneous NCPs [21,22]. The PVA consists of a carbon chain backbone comprising hydroxyl groups that can form hydrogen bonding. Such a characteristic of PVA allows for producing polymer NCPs quickly. The high transparency and prevention of oxygen transport of PVA make it a perfect candidate to be integrated into organic solar cell multilayer coatings [14].

Recently, NCPs with comparatively high refractive index polymers have been widely studied. It is well-known that organic materials are distinguished by transparency, light weight, cost-effectiveness, ease of processing, and good mechanical properties [23]. As previously stated, the polar groups on the backbone of hydrophilic polymers are sufficient to form relatively too stable homogeneous NCPs, owing to burly interactions between the polar groups and the fillers’ cations [24]. In the last fifteen years, the favorable properties of NS materials have been used in many fields of technology [25]. To study the nature of structure and bandgap (BG) energy, optical absorption spectra of the crystalline and noncrystalline materials are the best means. From the absorption spectra analysis, one can gain insight into the atomic vibration and electronic states from the low energy and the high energy parts, respectively, of the spectra [26]. Thus, the accurate measurement of an optical constant is exceptionally essential.

Hemalatha and Rukmani [27] reported the bandgap of PVA impregnated with various wt.% of synthesized CeO_2_ by combustion method. In their study, there is a lack of information about important optical parameters, such as refractive index and optical dielectric function. They are unable to specify precisely the type of electron transition. The present work aims to address modification in optical properties of PVA polymer by narrowing the optical BG energy and increasing the index of refraction utilizing the incorporation of CeO_2_ NPs. The response absorption coefficient, optical dielectric constant, and dispersion RI behavior of PVA/CeO_2_ NCPs films versus stimulus of various concentrations of CeO_2_ NPs will be investigated. Two crucial approaches were examined to determine the energy BG and specify the type of electron transition.

## 2. Experimental

### 2.1. Materials and Preparation of Nanocomposite Solid Polymer Films

#### Materials and Preparation of nanocomposites (NC) Solid Polymer Films

The used raw materials in the current work were PVA purchased from Sigma–Aldrich and the nanosize cerium oxide CeO_2_. The NC film, based on PVA, was prepared via solution cast methodology. Firstly, the dissolution of PVA powder was carried out in distilled water at 90 °C, and then the solution was stirred well with the aid of a magnetic stirrer for three h to form identical solutions. Various quantities of CeO_2_ (1, 3, 5, and 7 wt.%) NPs were added separately under vigorous stirring to this solution. The samples were coded as SPNC-0, SPNC-1, SPNC-2, SPNC-3, and SPNC-4 for the pure PVA and 1 wt.%, 3 wt.%, 5 wt.%, and 7 wt.% of CeO_2_-doped samples, respectively. A series of films were obtained from homogenous solutions by casting in four plastic Petri dishes and gaining solvent-free film at room temperature for two weeks. To make sure of dryness of the films, desiccators containing blue silica gel desiccant were used. Table 1 presents various quantities of the synthesized PVA-based NCPs.

### 2.2. Measurements

The recording of absorption spectra of the samples was performed using double beam UV–Vis–NIR spectrophotometer (Model: Lambda 25, Perkin Elmer, Melville, NY, USA).

The X-ray diffraction patterns of the samples were taken at room temperature using A D5000 X-ray diffractometer (Malvern Panalytical Ltd., Malvern, UK) working at 40 kV voltage and 45 mA current correspondingly. In this case the samples were scanned with a monochromatic beam of X-ray radiation (λ = 1.5406 Å) (Malvern Panalytical Ltd., Malvern, UK) and glancing angles (2θ) between 10 and 80 with a 0.05 step size.

## 3. Results and Discussion

### 3.1. X-ray Diffraction (XRD)

**Figure 1:**
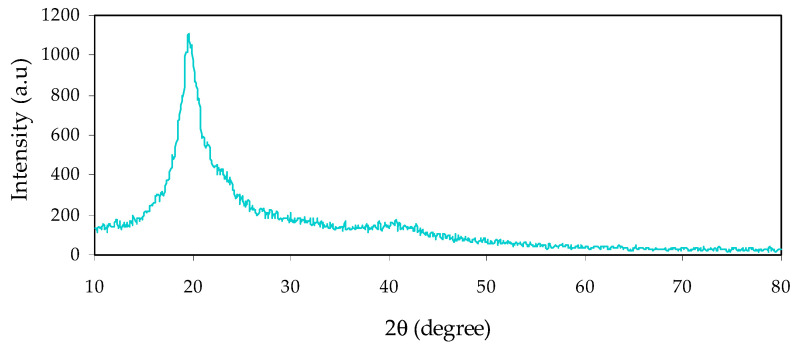
XRD pattern of neat PVA film.

**Figure 2:**
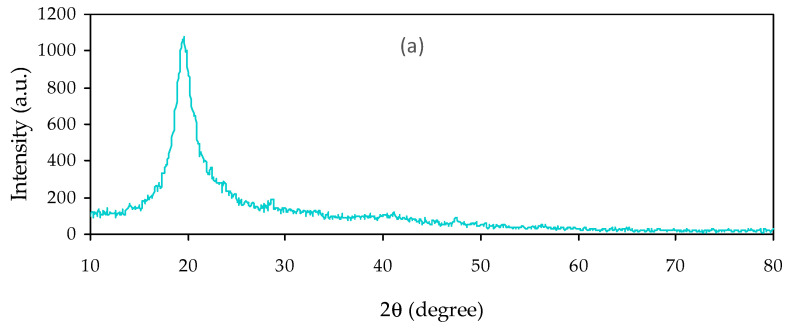

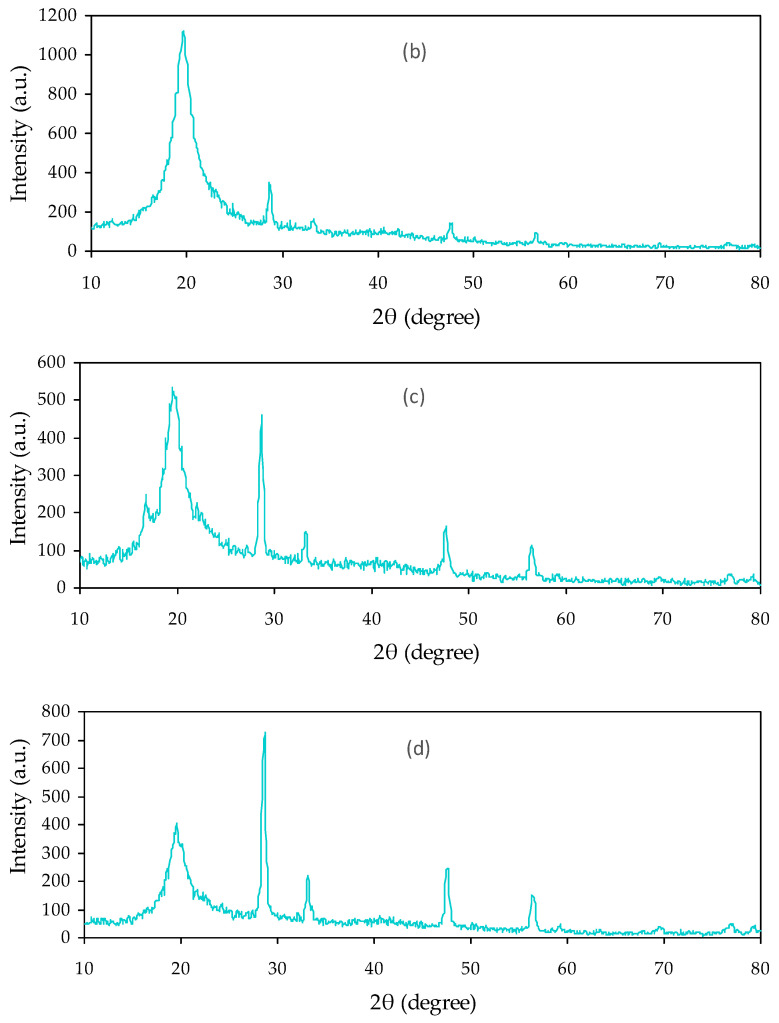
XRD pattern of (**a**) SPNC-1, (**b**) SPNC-2, (**c**) SPNC-3, and (**d**) SPNC-4 samples.

When ceramic filler or NPs were added into PVA, interactions presumably occurred with changing to some extent from the crystalline to amorphous polymer phases, improving physical properties. The most powerful technique to study the structural information of the polymer composites is XRD [6,28]. Figure 1 shows the XRD plot of neat PVA films. Previous study established that neat PVA exhibited characteristic peaks at 2θ value of 19.5° and shows the semicrystalline nature of PVA [29]. The intensity of the diffraction peak at 19.5° is relatively high, due to hydroxyl groups’ existence in the side chains, resulting in strong hydrogen bonding [18,30,31]. Figure 2a–d show the XRD pattern for PVA NCPs films. It is clear that the main peak of PVA at 19.5° is slightly broadened when the NCs are formed by the CeO_2_ addition, due to the crystalline phase’s disruption within PVA. The former study confirmed the impact of fillers’ addition that decreased the polymer’s diffraction peak intensity, due to its amorphous nature. In addition to essential peaks of PVA, several crystalline peaks appear with intensity increasing during increasing concentration of CeO_2_ NPs. The peaks of CeO_2_ are appeared in Figure 2a but with weak intensity, while obvious sharp peaks with high and low intensity can be distinguished in Figure 2c,d. The distinct peak positions centered at 2θ = 28.65°, 33.1°, 47.5°, 57.6°, 69.4° and 76.9° are characteristic peaks corresponding to the (111), (200), (220), (311), (222), (400), (331), (420), and (422) planes of CeO_2_ crystalline phases, separately [27,32,33]. The absence of peaks for other phases indicates the extent of the high purity of the product.

The fine crystalline index and single phase of CeO_2_ is the cubic structure [33]. It is proved that the nanometer-sized crystallites were formed, as evidenced from the broad peak. Scherrer equation can be used to determine the overall nanocrystalline size (D) [33],

(1) $$D=\;\frac{0.9\;\lambda }{\beta \;cos\theta }$$

where *λ* and *θ* refer to the X-ray wavelength and Bragg diffraction angle, respectively. β is full width at the half maximum (FWHM) of the peak. From the XRD pattern, for the main peak of CeO_2_ at 2θ = 28.65°, which is observable for all weight ratios of CeO_2_, the crystallite size was calculated using Equation (1) and presented in Table 2. Clearly, at high concentration of CeO_2_, the sharpness of crystalline peaks increased, and their width decreased, which is an evidence for the growth of CeO_2_ grain sizes (large crystallite size) [27].

### 3.2. Optical Properties

#### 3.2.1. Absorption Study

**Figure 3:**
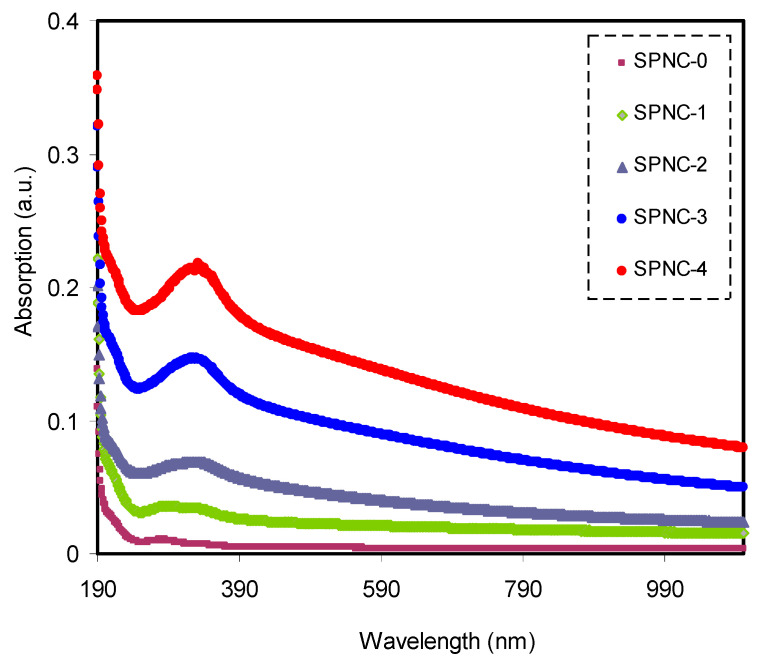
Absorption enhancement in doped samples compared to pure PVA.

Dealing with optical properties and recognizing electronic transitions within polymer NC films can be carried out using ultraviolet–visible (UV–Vis) spectroscopy. The optical absorption can be increased upon the addition of CeO_2_ filler into PVA samples, as shown in Figure 3. This can be interconnected to hosting new electronic levels into the prohibited gap within the PVA polymer [8,34,35]; thereby, the wide BG of PVA can be narrowed. The influence of CeO_2_ filler on the pure PVA can be confirmed from the shifting of the peak in the UV–Vis Spectra short toward longer wavelengths for all polymer samples encompassing CeO_2_ filler. A considerable absorption occurs at high wavelengths, as exhibited in Figure 3. It was also noticed that the absorption occupied a wide range of the spectra, suggesting the eligibility of these samples as raw materials for utilization in optoelectronic devices. The stronger exponential performance of the absorption vs. wavelength is evidenced for the NCPs films, compared to pure PVA.

**Figure 4:**
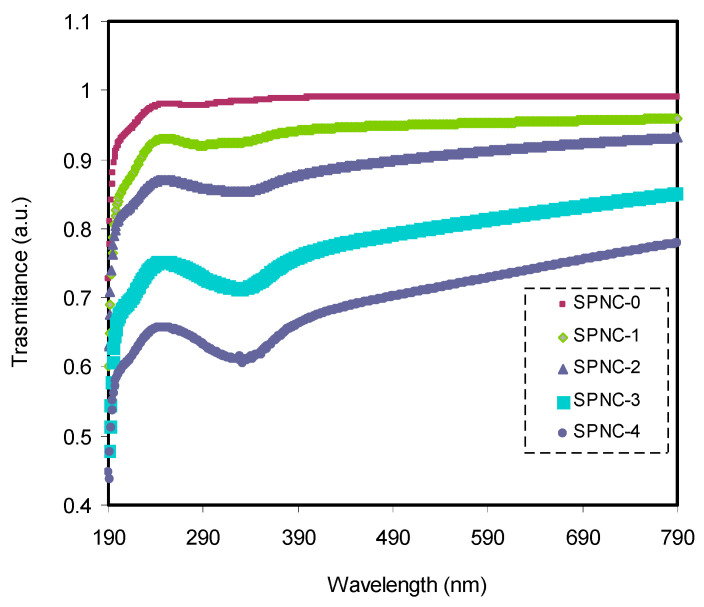
Variation in transmittance spectra for pure and CeO_2_-doped samples.

Figure 4 displays the transmittance of pure and doped PVA samples with varying amounts of CeO_2_. The pure PVA had a relatively high degree of transparency beyond the visible region, over 97%, although the transparency decreased below the visible region due to the high absorption of the films. The transparency lowered and reached almost 74% for the doped PVA with CeO_2_ at 7%. This transparency drop aroused from relatively high refractive index and significant scattering of films at high CeO_2_ content. In the doped films, the spectrum possesses a shoulder, while in the pure PVA, a weak intensity shoulder can be seen. This suggests that the functional groups inside the polymer chains interact strongly with the added CeO_2_ fillers, causing the doped films’ transparency to decrease.

**Figure 5:**
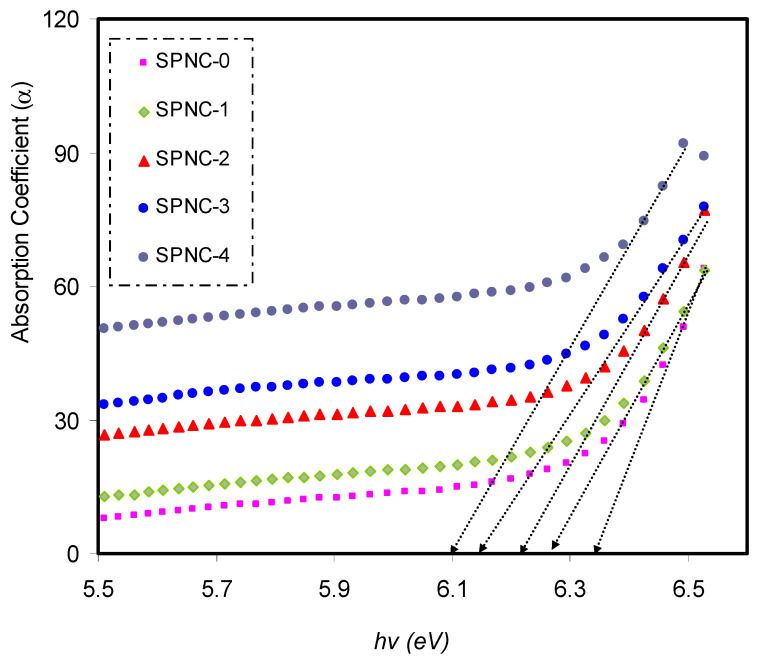
Shift in absorption coefficient (α) for CeO_2_-doped samples relative to the pure sample.

Identifying the type of electron transitions in materials can be determined from the alterations in absorption spectra. Band-to-band transitions are associated with fundamental absorption, taking the form of a fast rise in absorption called the absorption edge. This edge is a measure of the optical BG energy [36,37]. The extent of absorption is based on an absorption coefficient *α* (*υ*), indicating the relative rate at which the incident light intensity attenuates in a unit length of the medium [11,38,39]. Determination of the (*υ*) at the equivalent frequency (υ) from the absorbance spectra *A*(*υ*) can be performed using Beer–Lambert law [6,36,37]:

(2) $$\alpha \left(v\right)=\frac{2.303}{d}\log\left(\frac{I_{o}}{I}\right)=\frac{2.303}{d}A\left(v\right)$$

where the incident light and transmitted light intensities are denoted by *I_o_* and *I,* respectively, and the sample thickness is represented by *d.* Figure 5 shows *α*(*υ*) against photon energy (*hυ*) for pure PVA and PVA films doped with CeO_2_ samples. The absorption edge values can be calculated by extrapolating the linear part to zero of the ordinate. Upon the addition of CeO_2_ NPs, the absorption edge was found to shift to the direction of low photon energy, from 6.38 eV to 6 eV for pure PVA and PVA NC samples. Alteration in the sample band structure points out from the drop in the absorption edge value as CeO_2_ added, resulting from the establishment of fresh localized states in the mobility gap [40]. The absorption edge values of the pure and CeO_2_ nanofiller-doped samples are tabulated in Table 3.

#### 3.2.2. Complex Optical Dielectric Constant ($\varepsilon_{r}$ and $\varepsilon_{i}$) and Refractive Index (n) Study

**Figure 6:**
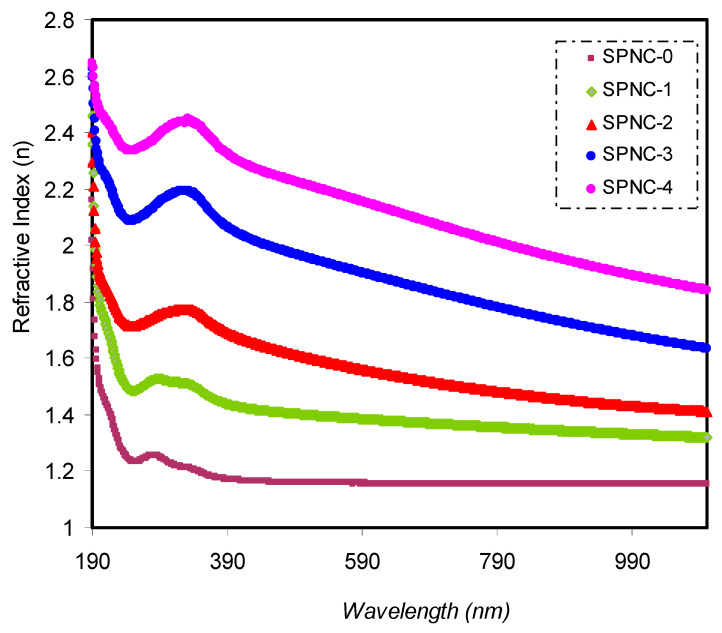
Increased refractive index (n) of the CeO_2_-doped samples relative to the pure PVA sample over a wide range of wavelength.

Figure 6 indicates the ***n*** of PVA with varying concentrations of CeO_2_ fillers. From the absorbance (A) and reflectance (R) spectra, several optical parameters of the samples can be derived. The optical ***n***, which measures the light speed’s reduction rate through a medium, is essential among these parameters [41]. For the calculation of the sample refractive index value, the reflectance (*R*) and extinction coefficient (*K*) are taken into account; the following equation is applied [42]:

(3) $$\;n=\;\left[\frac{\left(1+R\right)}{\left(1-R\right)}\right]+\sqrt{\frac{4\times R}{{\left(1-R\right)}^{2}}}-K^{2}$$

The increment in CeO_2_ NPs is contributing to an increase in the refractive index. It is shown that a broad dispersion region and a high refractive index over a wide spectrum of wavelengths characterize the samples. The high ***n*** polymers have recently been used for several applications, such as display devices, advanced organic light-emitting diodes (OLEDs), anti-refractive coatings, and various semiconductors [43,44]. Based on this impressive property, numerous researchers have focused on increasing the ***n*** of polymers. Numerous methodologies have been implemented, for instance, incorporation of perfluorocyclobutane group, adamantane group, and sulfur atom into polymers and organic/inorganic DPs [45,46]. The refractive index is the essential property of those materials utilized in optoelectronic and photonic devices.

One such in particular are organic solar cells (OSCs). Thus, a deep understanding of this property is of great importance to evaluate efficient solar cells [47]. It is well-known that a high refractive index is requested for organic solar cells at a large scale [48,49,50,51]. In the present work, the obtained refractive index for PVA by ion incorporation is relatively higher than reported throughout the literature [49]. The broad dispersion of *n* for PVA by ion incorporation is more significant than that reported by Shi et al. for nonlinear optical (NLO) polymers, polyetherketone (PEK-c) [50]. This is a vital material for the large-scale applications of integrated-optic devices, such as electro-optic modulators and switches. The refractive index for a variety of polymer composites is presented in Table 4.

**Figure 7:**
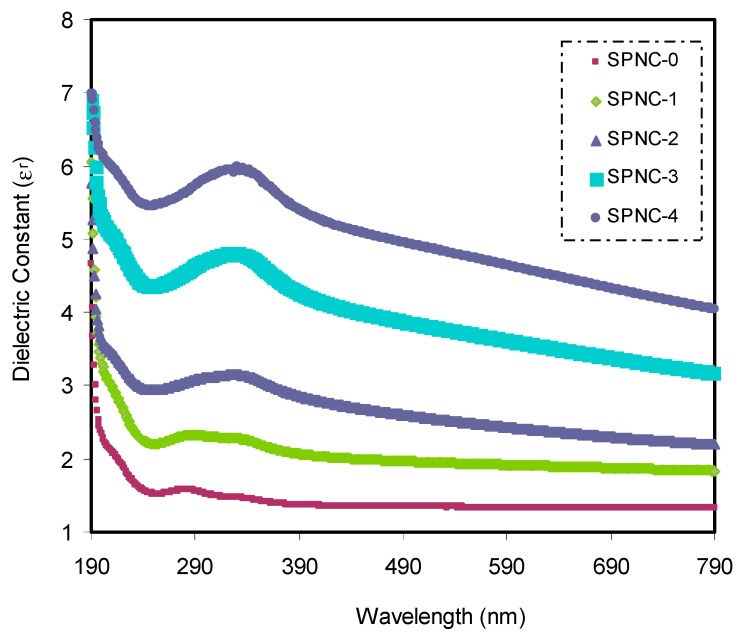
Dielectric constant spectra as a function of wavelength for pure and CeO_2_-doped PVA samples.

The complex optical dielectric constant consisting of real ($\varepsilon_{r}=n^{2}-k^{2}$) and imaginary ($\varepsilon_{i}=2nk$) parts has also been acquired. Several parameters can be derived from this spectrum, closely associated with the energy state density within the samples’ optical BG [55,56]. It should be noted that the dielectric function cannot be measured from optical spectroscopy, directly. However, direct functions, such as the R, A, ***n***, and extinction coefficient (*K*), can be accessed. Figure 7 shows the *ε**_r_*** of the PVA-based NCPs samples. The dielectric function (DF) largely relies on the materials’ band structure. The analysis of DF using optical spectroscopy has been established to be informative in imagining the materials’ overall band structure [57,58]. It is shown from Figure 7 that the ***ε_r_*** increases with the quantity of CeO_2_ NPs are increasing. This rise in dielectric constant suggests that the energy density of states is comparatively high. This also results in a polarization boost, which gives a relatively high dielectric constant value. The optical ***ε_r_*** is seen as a dispersion region at low wavelengths, while the values are almost constant at high wavelengths.

Due to exposure to an incident electromagnetic light, materials’ internal charge structure undergoes a time-varying force. This is owing to the influence of the electric field component of the incident wave. The broad dispersion region is correlated with the polar nature of the samples. It is worth noting that, due to its inertia at high wavelengths, polar molecules cannot obey the field alternation [59]. The dispersion in dielectric constant shows that these materials can be qualified for industrial use, particularly in designing optical devices and the optical communication field [60,61,62].

#### 3.2.3. Bandgap Study

It is well-known that optical properties and the electronic band structure are closely correlated [13,63,64]. One of the characteristics of absorbing isotropic materials is the complex optical DF dependence on the wavelength (*λ*) [65]. The decisive optical functions can be determined from both the real and imaginary parts of the DF [66]. More precisely, the electronic polarizability and the electronic absorption can be determined from the *ε_r_*_(ω)_ and the *ε_i_*_(ω)_, correspondingly [67].

The direct and indirect BG transitions are both related to the ε_(ω)_, while direct transitions are more involved in the *ε*_(ω)_, in comparison to the indirect transitions, as a consequence of mediation by phonons in the latter one [64]. It was previously verified that it is difficult to accurately determine the optical BG from the KT and Taucs equations from certain disposable constants in formulas [68,69,70,71]. However, a simple and effective technique has been established with sufficient accuracy to analyze the optical BG [6,13,70]. The desired technique to determine the optical BG precisely is data analysis of optical dielectric loss. Such a technique is established on the modern improvements in quantum mechanics and numerous models. Based on quantum mechanics, the time-dependent perturbations of ground electronic state are the foundation in the photon–electron interaction characterization inside a system. The photon absorption and emission are causes of the alteration from full to vacant states. The dielectric function’s imaginary part ε_i(ω)_ has the following form [6]:

(4) $$\varepsilon_{2}=\frac{2\pi e^{2}}{\Omega \varepsilon_{o}}\underset{K,V,C}{\sum }{\left|\langle \Psi_{K}^{C}\left|u.r\right|\Psi_{K}^{V}\rangle \right|}^{2}\delta \left(E_{K}^{C}-E_{K}^{V}-E\right)$$

where *u* and *k* are the vectors that characterize the incident electric field polarization and the reciprocal lattice, respectively. The superscripts *C* and *V* signify the conduction and the valence bands, respectively, and *ω* symbolizes the incident photon frequency [72].

**Figure 8:**
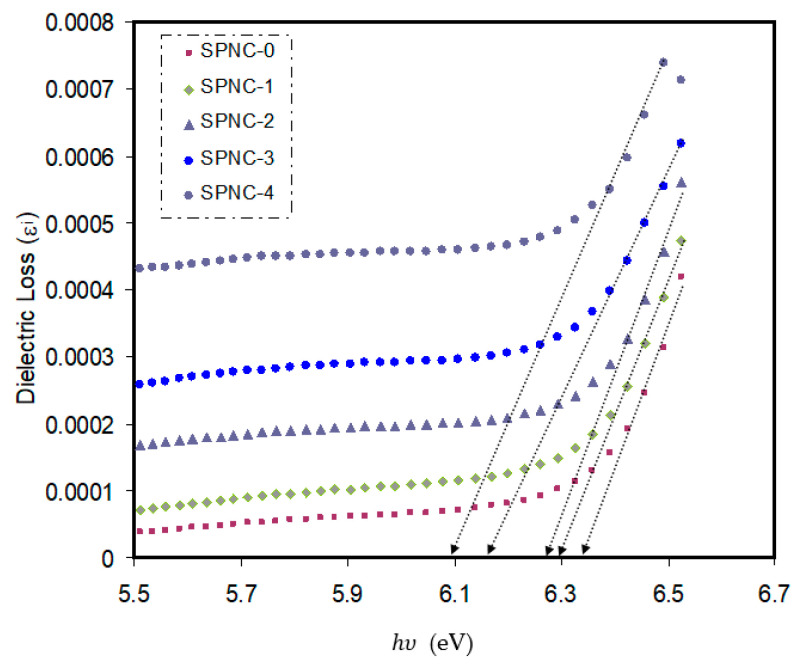
Variation in dielectric loss as a function of photon energy for pure and CeO_2_-doped PVA samples.

Figure 8 displays the imaginary part of the dielectric constant *ε_i(ω)_* spectra. From the joint of linear parts on the *x*-axis, one can determine the optical BG. It is important to note that the optical spectra result from the top of full bands to the lowest of conduction bands. The fundamental absorption edge, also referred to as the basic absorption edge, is the most distinctive optical BG feature [73]. It is noted that the significant points are closely correlated with the values of the BG [74].

Table 5 shows the BG values obtained from dielectric loss spectra. Both the energy BG and the refractive index of CeO_2_ NPs do not reflect the same effect. The optical properties, i.e., refractive index and energy gap, have been reported to be closely interrelated to the variation in material compositions and atomic configurations [75]. Within the pure PVA BG, the formation of localized states, i.e., rising density state, effectively decreases the BG energy. The high refractive index of the samples, in other words, means the high density of state. From the renowned Clausius–Mossotti relation, the refractive index’s reliance on film density can be rationalized [57].

The type of electronic transition inside the material BG can be recognized using the interband absorption model. A photon can excite an electron from a top, occupied valence band to a bottom, unoccupied conduction band. There is a mathematical relationship based on this model, from which the material’s optical BG can be determined [76,77]:

(5) $$\;\alpha h\nu =B{\left(h\nu -E_{g}\right)}^{P}$$

where *B* and *E_g_*, respectively, signify the energy-independent constant and optical energy BG. The constant *p* specifies the nature of the optical transition from the valence- to conduction-band, i.e., fundamental absorption.

**Figure 9:**
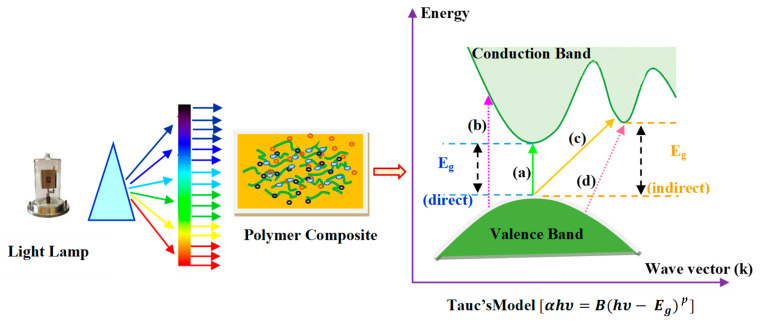
Illustration of possible electronic transitions (a) direct allowed, (b) direct forbidden, (c) indirect allowed, and (d) indirect forbidden [44,59].

According to the selection rule, when *p* = 1/2, it indicates the allowance of direct transition. The electron vertically transfers from the top valence band to the bottom conduction band during the direct allowance transition. On the other hand, the nonvertical transitions are usually known as indirect allowed transitions, and *p* takes a value of 2 in this case [76]. It should also be noted that *p* takes 3 and 3/2 values for indirect and direct forbidden transitions, respectively. Based on the Taucs model, the different kinds of electronic transitions between the valence band (VB) and conduction band (CB) can be shown in Figure 9 [59].

**Figure 10:**
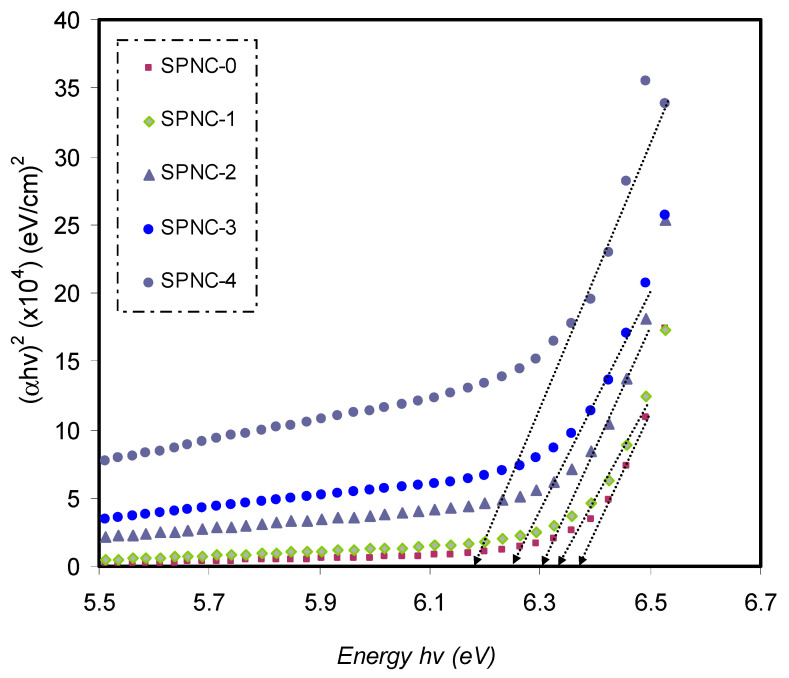
The plots of ${\left(\alpha h\upsilon \right)}^{2}$ versus (*hυ*) for pure PVA and PVA-doped samples.

The dependence of ${\left(\alpha h\upsilon \right)}^{2}$ versus hυ of pure PVA and PVA-doped samples are exhibited in Figure 10. The direct energy gaps of all the samples are presented in Table 5. The optical energy gap values can be obtained from extrapolation of the linear part of the plot to zero absorption. From Table 5, one can note that the values of the optical bandgaps calculated from dielectric study are close to those obtained from Tauc’s relation, where (*p* = 1/2). This outcome reveals that the prepared samples exhibi the direct allowed transition, which means that the samples are having direct optical BG.

It should be noticed that the energy BG of pure PVA, in comparison with other samples, is relatively large (5.38 eV). In the present work, the pure PVA’s obtained direct optical BG is well-matched by the literature [78]. Based on solid-state physics principles, the energy BG in a solid is a range of energy where no electron states exist. The energy required to free a bound electron from the outermost shell to become a conductive electron within the materials is the energy BG. Furthermore, in terms of electrical conductivity, the BG energy is considered the main barrier in solid materials. Materials with small and large BG are known as insulator and semiconductor materials, respectively. In this regard, the pure PVA is a pure insulator, since its BG is 5.36 eV large, suggesting the lack of free carrier absorption. In PVA, the only existing band is the interband transitions that becoming interesting material with relatively high photon energy, as shown in Figure 8a.

In the present work, the BG of PVA can be changed to be eligible for a specific application via the addition of an optimum portion of the tea solution. It has been emphasized that the narrow BG materials are considered as the desired candidate materials to compensate for the low energy photon harvest [79]. Thus, the process of lowering the BG energy of PVA polymer via the incorporation of environmentally friendly tea materials can be regarded as a breakthrough in optical materials. It also makes a new revolution in organic solar cells and optoelectronic devices.

**Figure 11:**
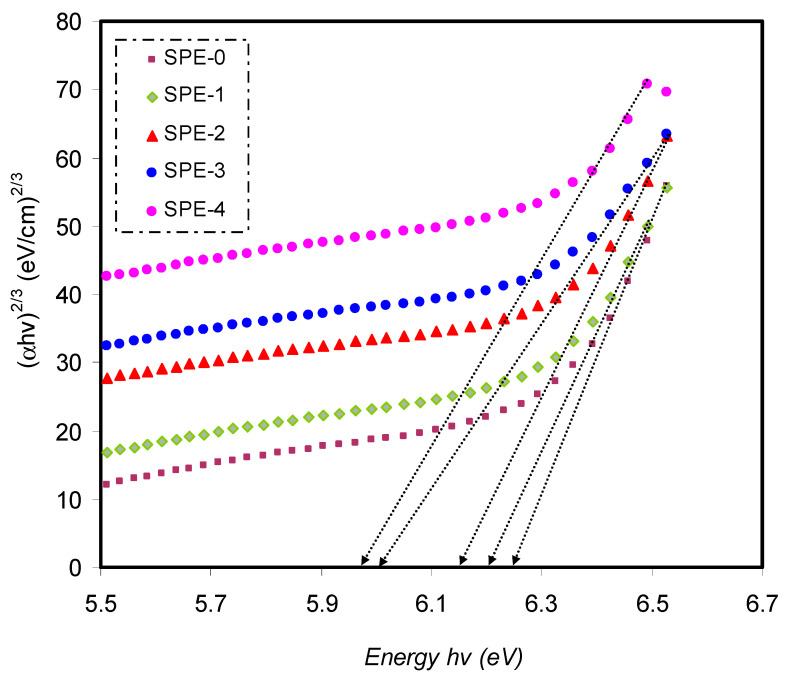
The plots of ${\left(\alpha h\upsilon \right)}^{2/3}$ versus (*hυ*) for pure PVA and PVA-doped samples.

**Figure 12:**
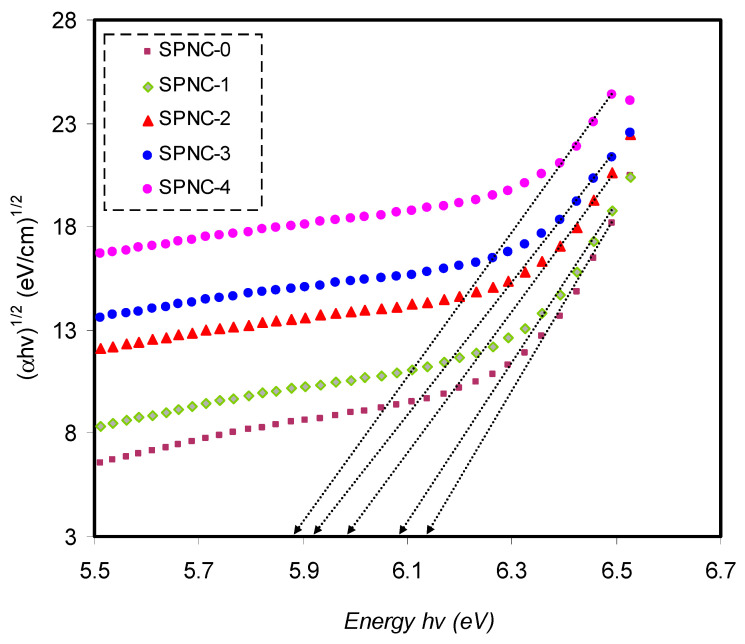
The plots of ${\left(\alpha h\upsilon \right)}^{1/2}$ versus (*hυ*) for pure PVA and PVA-doped samples.

Close the ultimate band edge, the direct and indirect transitions will occur and can be shown by plotting (*αhυ*)^1/2^ and (*αhυ*)^2^, dependent on photon energy (*hυ*) [80,81]. Figure 10, Figure 11 and Figure 12 demonstrate the plot of (*αhυ*) versus photon energy (*hυ*) for each exponent (*n*) value in the Tauc’s equation. In these plots, linear parts can be seen, and the BG energies can be determined from the intersections of the *x*-axis. Amorphous materials’ absorption edge can be evaluated from the indirectly allowed transitions, depending on their electronic configuration [82]. Thus, in the case of amorphous materials, indirect transitions should be taken into account.

On the other hand, in the indirect BG materials, the electron transition from the valence to conduction bands correlates with a phonon of right crystal momentum magnitude [80]. Since the acquired optical BG values differ according to the exponent (*n*), choosing (*n*) value can be principally impossible. Therefore, based on Equation (4), the type of conduction mechanism can be rationalized. In the meanwhile, an additional parameter, namely the dielectric constant from the imaginary part (*ε*_2_), is used to calculate the actual *E_opt_*; thereby, the exact exponent value can be chosen [82].

#### 3.2.4. Wemple–DiDomenico (W–D) Model

Refractive index and its dispersion manners are among the decisive properties of the optical materials. Particularly, the refractive index dispersion is found to be very significant for optical communication and in designing devices for spectral dispersion [83]. The refractive index dispersion in the normal region can be explored using the well-known single oscillator model presented by W–D [84,85]. The investigation is usually done by introducing a dispersion energy parameter (*E_d_*) as a gauge of the force of interband optical transition. Both the coordination number with the charge allocation in each unit cell are combined through the *E_d_* parameter, which is strongly interrelated to the chemical bonding [86,87]. Nevertheless, a single oscillator parameter (*E_o_*) is proportional to the energy of oscillator (average energy bandgap). Using a semiempirical equation (Equation (6)), both photon energy and refractive index under the interband absorption edge can be correlated as follows:

(6) $$n^{2\;}-1=\frac{E_{d}\;E_{o}}{\left[E_{o}{}^{2}-{\left(hv\right)}^{2}\right]}$$

**Figure 13:**
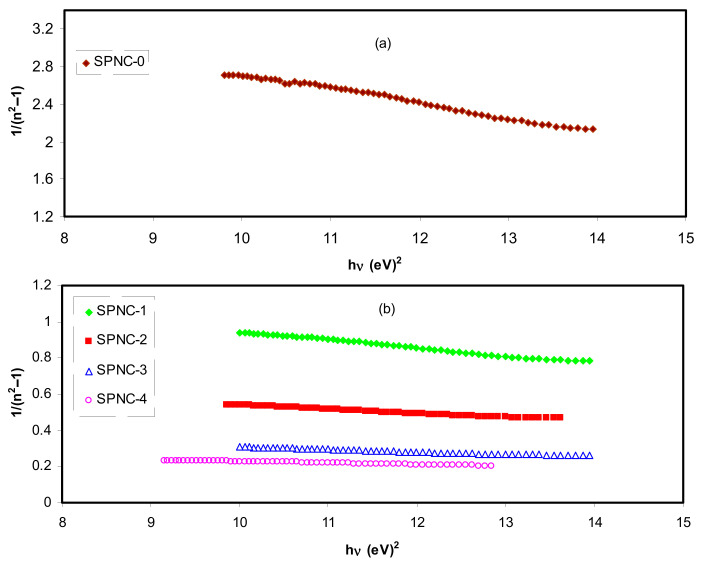
Variation of (n^2^ − 1)^1/2^ versus (hυ) for (**a**) pure PVA film and (**b**) PVA NCPs films.

As portrayed in Figure 13, the data on the plots of $1/(n^{2}-1)$ against *(hυ)*^2^ were fitted with linear regression lines to acquire the values of *E_d_* and *E_o_* from the intercept and slope, correspondingly. The calculated values of the *E_o_* and *E_d_* are presented in Table 6. A raise in *E_d_* (see Table 5) is observed, which characterizes the increase of average strength of interband optical transitions. The *n_o_* value achieved from the W–D model is sufficiently close to *n* values shown in Figure 6 at high wavelengths. For the present films, empirically the *E_o_* values are near to the *E_g_* values presented in Table 5. Hence, the from W–D model, it is achievable to get the *E_g_* and ($n_{o})$ values for the films; however, it is still hard to identify the type of transition from VB to CB.

## 4. Conclusions

In this study, the solution casting method was used to prepare PVA/CeO_2_ NC polymer films. The XRD pattern of the PVA film exhibited a small peak broadening with increased amorphous nature upon the addition of the CeO_2_, which signifies interaction between the host polymer and the nanofiller. At high CeO_2_ content, some crystalline peaks with the average crystalline size of 11 nm were observed, which are related to the CeO_2_ NPs crystalline phases. It is noticed from the optical study that the CeO_2_ NPs significantly improved the optical absorption of PVA film by introducing new electronic states into the forbidden gap of the host polymer. This feature can play a vital role in enabling PVA polymer for future optoelectronics applications. Accordingly, the transparency of pure PVA film was dropped from 97% to 74% with the addition of 7 wt.% of CeO_2_ nanofiller in the visible region. Additionally, the small hump that appeared in the doped films’ absorption spectra was a clear sign for good interaction between the functional groups of the host polymer and the inserted filler. The doped samples showed a broad dispersion and high refractive indexes over a wide range of wavelengths, ascribed to the increase of charge density. Moreover, the rise in dielectric constant with wide dispersion upon the insertion of larger CeO_2_ NPs revealed the high population of states’ energy density, which is favorable for many optical devices. Ultimately, both Tauc’s model and optical dielectric loss (ε”) parameters were used to calculate the optical BG. Thus, the optical dielectric loss study can be considered as an effective method to determine the type of electronic transition and compute the optical BG. The single-oscillator energy (*E_o_*) and the dispersion energy (*E_d_*), which characterize the average strength of interband optical transitions, were successfully identified using Wemple–DiDomenico single oscillator model.

## Figures and Tables

**Table 1:** Sample designation and CeO_2_ concentration in the prepared poly (vinyl alcohol) (PVA)-based nanocomposites (NCPs).

Sample Designations	CeO_2_ Nanofiller Concentration (wt.%)
SPNC-0	0
SPNC-1	1
SPNC-2	3
SPNC-3	5
SPNC-4	7

**Table 2:** Crystallite size calculation for CeO_2_ at various concentrations.

Sample	*D* (nm)
SPNC-1	13.701
SPNC-2	16.442
SPNC-3	20.55
SPNC-4	23.489

**Table 3:** Values of absorption edge for the whole prepared samples.

Sample Designations	Absorption Edge (eV)
SPNC-0	6.34
SPNC-1	6.28
SPNC-2	6.21
SPNC-3	6.14
SPNC-4	6.09

**Table 4:** Refractive index values of various polymer composite systems.

Composition	Refractive Index	λ (nm)	Ref.
PVA: 5 wt.% Al	2.14	1100	[39]
PGMA: 60 wt.% TiO_2_	1.8	800	[52]
PEO: 4 wt.% SnTiO_3_	2.47	1100	[53]
PMMA: 25 wt.%ZnO	~1.65	400	[54]
PVA:PVP:0.03 mole Ag_2_S	1.52	1100	[13]
PS: 8 wt.% SnTiO_3_	2.6	1100	[44]
PVA:7 wt.% CeO_2_	1.93	**1100**	**Present work**

**Table 5:** Estimated optical bandgap from Taucs method [${\left(\alpha h\upsilon \right)}^{\frac{1}{p}}$ versus hυ] and *ε_i_* plot.

Sample Code	*E_g_* for *p* = 1/2	*E_g_* for *p* = 3/2	*E_g_* for *p* = 2	*E_g_* from *ε_i_* Plot
SPNC-0	6.39	6.25	6.14	6.34
SPNC-1	6.33	6.2	6.09	6.29
SPNC-2	6.3	6.15	5.98	6.27
SPNC-3	6.25	6	5.93	6.18
SPNC-4	6.18	5.97	5.88	6.09

**Table 6:** Various parameters calculated based on W–D single oscillator model.

Sample Code	*E_d_*	*E_o_*	*n_o_*
SPNC-0	1.223	5.24675	1.111
SPNC-1	4.051	5.610461	1.312
SPNC-2	7.738	5.874444	1.522
SPNC-3	13.621	5.87333	1.821
SPNC-4	19.247	6.041523	2.046

## Data Availability

No new data were created or analyzed in this study. Data sharing is not applicable to this article.
